# Expression of the RNA Helicase DDX3 and the Hypoxia Response in Breast Cancer

**DOI:** 10.1371/journal.pone.0063548

**Published:** 2013-05-16

**Authors:** Guus M. Bol, Venu Raman, Petra van der Groep, Jeroen F. Vermeulen, Arvind H. Patel, Elsken van der Wall, Paul J. van Diest

**Affiliations:** 1 Departments of Pathology, University Medical Center Utrecht Cancer Center, Utrecht, The Netherlands; 2 Division of Internal Medicine and Dermatology, University Medical Center Utrecht Cancer Center, Utrecht, The Netherlands; 3 Department of Radiology and Radiological Science, Johns Hopkins University School of Medicine, Baltimore, Maryland, United States of America; 4 Department of Oncology, Johns Hopkins University School of Medicine, Baltimore, Maryland, United States of America; 5 MRC, University of Glasgow Centre for Virus Research, Glasgow, United Kingdom; National University of Ireland Galway, Ireland

## Abstract

**Aims:**

DDX3 is an RNA helicase that has antiapoptotic properties, and promotes proliferation and transformation. In addition, DDX3 was shown to be a direct downstream target of HIF-1α (the master regulatory of the hypoxia response) in breast cancer cell lines. However, the relation between DDX3 and hypoxia has not been addressed in human tumors. In this paper, we studied the relation between DDX3 and the hypoxic responsive proteins in human breast cancer.

**Methods and Results:**

DDX3 expression was investigated by immunohistochemistry in breast cancer in comparison with hypoxia related proteins HIF-1α, GLUT1, CAIX, EGFR, HER2, Akt1, FOXO4, p53, ERα, COMMD1, FER kinase, PIN1, E-cadherin, p21, p27, Transferrin receptor, FOXO3A, c-Met and Notch1. DDX3 was overexpressed in 127 of 366 breast cancer patients, and was correlated with overexpression of HIF-1α and its downstream genes CAIX and GLUT1. Moreover, DDX3 expression correlated with hypoxia-related proteins EGFR, HER2, FOXO4, ERα and c-Met in a HIF-1α dependent fashion, and with COMMD1, FER kinase, Akt1, E-cadherin, TfR and FOXO3A independent of HIF-1α.

**Conclusions:**

In invasive breast cancer, expression of DDX3 was correlated with overexpression of HIF-1α and many other hypoxia related proteins, pointing to a distinct role for DDX3 under hypoxic conditions and supporting the oncogenic role of DDX3 which could have clinical implication for current development of DDX3 inhibitors.

## Introduction

In the Western world, one in eight women will develop breast cancer during their life and breast cancer is causing about 458.000 deaths worldwide per year [1], [2]. Aggressive forms of breast cancer are frequently refractory to treatment [3], even to established targeted therapy, and thus have a high risk of relapse and formation of distant metastases [4]. Identification of molecular pathways involved in aggressive forms of breast cancer is therefore important to design novel targeted therapeutic agents to counteract tumor progression and metastasis.

DDX3, also known as DDX3X because of its location on the X chromosome, is a member of the DEAD-box RNA helicase family which is involved in transcription, RNA splicing, nuclear export of mRNA and translation initiation [5], [6]. Initially, DDX3 was studied because of its manipulation by viruses like hepatitis C (HCV) and human immunodeficiency virus (HIV) [7], [8]. Recently DDX3 has been associated with cancer [9]. Conflicting evidence exists with regard to its tumor enhancing or repressing properties. Nevertheless, DDX3 was proven to have antiapoptotic properties [10], [11], promotes proliferation and cellular transformation[9], [12]–[14]. Recently, novel compounds were developed which could potentially inhibit DDX3 activity[15]–[20].

A recent *in vitro* study [21] showed that DDX3 is a direct downstream target of HIF-1α, the predominant factor in the mammalian hypoxia response [22]. Hypoxia is an important event in breast carcinogenesis[23]–[26], causing a more aggressive phenotype with increased invasiveness and proliferation, formation of metastases, resistance to therapy [27] and poorer survival [28], [29].

However, no data are yet available on the relation between DDX3 and hypoxia in human breast cancer, or any other human tumors specimens. Therefore, we set out to correlate expression of DDX3 and HIF-1α in a large set of human invasive breast cancers. Furthermore, we correlated DDX3 expression to expression of various other proteins upstream of HIF-1α like EGFR [30], HER2 [31], Akt1[32]–[34], p53[35]–[39], COMMD1 [40], [41], FER kinase [42], PIN1 [43] and FOXO4 [44]. Also we assessed proteins downstream of HIF-1α such as ERα [45], [46] Transferrin receptor (TfR) [47], FOXO3A [48] and Notch1 [49], [50]. Finally, we included proteins that have been associated with HIF-1α without clear functional relationship like E-cadherin [51], p21 [52], c-Met [53], [54] and p27 [55].

## Materials and Methods

### Patients

Representative paraffin embedded tissue blocks of 422 breast cancer patients collected between 2004 and 2007 were taken from the archive of the Department of Pathology of the University Medical Centre in Utrecht and routinely processed to four tissue microarrays (TMA) as described before [56], [57].

Clinicopathological data including tumor stage, histological data (type, grade, mitotic index (MAI), estrogen receptor alpha (ERα) and human epidermal growth factor receptor 2 (HER2)) status was collected from patient files (Table 1). Protein expression data by immunohistochemistry of HIF-1α, FOXO3A, FOXO4, PIN1, Akt1, COMMD1, p53, p21, p27, EGFR, E-cadherin, GLUT1 and CAIX was derived from previous studies[34], [40], [58]–[62].

**Table 1:** Patient characteristics.

		*N* (422)						missing			
Mean age (range)		61.0			(28–88)			0			
Tumor size											
≤20 mm		212			50%			3			
≤50 mm		181			43%						
>50 mm		26			6%						
Lymph node status											
Positive*		193			48%			18			
Negative**		211			52%						
Histological type											
ductal		343			82%			1			
lobular		42			10%						
other		36			9%						
Grade											
I		80			20%			26			
II		145			37%						
III		171			43%						
Mitotic index (range)	17.2			(0–196)			0				
Estrogen receptor#											
Positive		335			79%			0			
Negative		87			21%						
Progesterone receptor#											
Positive		247			59%			1			
Negative		174			41%						
HER2 receptor											
Positive		44			10%			0			
Negative		378			90%						

* Positive = ≥N1mi.

** Negative = N0 or N0(i+) (according to TNM 7^th^ edition, 2010).

# 10% cut-off.

Use of anonymous or coded left over material for scientific purposes is part of the standard treatment contract with patients in the UMCU [63].

### Immunohistochemistry

Sections of 4 µm were cut, mounted on SuperFrost slides (Menzel&Glaeser, Brunswick, Germany), deparaffinized and rehydrated. Endogenous peroxidase was then blocked for 15 min with a buffer solution containing 0.3% hydrogenperoxide. Antigens were retrieved by boiling for 20 min in 10 mM citrate buffer (pH 6.0) (for DDX3, c-Met, TfR, FER kinase and Notch1), cooled and washed in PBS. Nonspecific binding sites were blocked with a 2% normal goat serum, 1% BSA in PBS (pH 7.4) (Notch1). TMAs were subsequently incubated in a humidified chamber for 1 hour with polyclonal rabbit anti-DDX3 R648 [64] diluted 1∶1000, TfR 1∶300 (13–6800, Invitrogen, Breda, The Netherlands) and FER kinase 1∶300 (clone 5D2, Cell Signaling Technologies, USA). Primary antibodies against c-Met 1∶100 (18-2257, Zymed, Invitrogen) and Notch1 1:100 (Cell Signaling Technologies, USA) were incubated overnight at 4°C. Subsequently, sections were washed in PBS and incubated for 30 min with secondary antibodies (Brightvision, Immunologic, Duiven, The Netherlands) washed with PBS and developed with diaminobenzidine. Slides were counterstained with hematoxylin, dehydrated and cover-slipped. Appropriate positive and negative controls were used throughout.

### Scoring of Immunohistochemistry

Scoring was done by a single experienced pathologist (PJvD). Intensity of cytoplasmic DDX3, FER kinase and membranous E-cadherin, TfR and c-Met was scored semi-quantitatively from 0–3 and percentages of cells with nuclear DDX3 and Notch1 expression were estimated. Out of three cores from the same patient, the maximum cytoplasmic DDX3 score was used for further analysis.

DDX3 scores 1 and 2 were grouped as low DDX3 expression and evaluated against high DDX3 expression (scores 3). For E-cadherin, TfR, c-Met and FER kinase scores 0 and 1 were defined as low expression versus score 2 and 3 as high expression. For HIF-1α, the 1% threshold was used as before [59].

### Statistics

Expression levels of DDX3 and the other proteins were compared by chi-square test or t-test whenever applicable. Logistic regression or ANCOVA was used for multivariate analysis to determine dependence of these relations on HIF-1α.

Since EGFR and HER2 are upstream regulators of HIF-1α via PI-3K/AKT, we also assessed the relation of EGFR and HER2 with DDX3 independent of Akt1 and HIF-1α. In lobular breast cancer there is very little or no expression of E-cadherin, so the lobular cancers were excluded in analysis with respect to E-cadherin.

Pearson correlation coefficient was determined for correlation analysis.

All statistical analyses were carried out with SPSS 17.0 for Windows. (SPSS Inc., Chicago, IL, USA), regarding two-sided p-values below 0.05 as significant.

## Results

DDX3 staining could be evaluated in 366 of the 422 breast cancer cases. The drop outs were caused by damaged or detached cores during cutting, mounting, or staining, or did not contain tumor. All breast cancer cases showed some expression of DDX3 of which 127 (35%) showed strong cytoplasmic DDX3 expression.(table 2).

**Table 2:** Expression of DDX3 in relation to oxygen sensing proteins.

			Cytoplasmic DDX3				Multivariate	
		*N* (%)	Low (%)	High (%)	OR	p value^a^	OR	p value^b^
		366	239	127				
HIF-1α	≤1%	214 (66)	155 (75)	59 (51)	2.83	<0.001	2.52	0.001
	>1%	108 (34)	52 (25)	56 (49)				
GLUT1	negative	123 (39)	94 (46)	29 (27)	2.36	0.001	1.94	0.021
	positive	190 (61)	110 (54)	80 (73)				
CAIX	negative	62 (19)	49 (24)	13 (11)	2.39	0.012	2.23	0.042
	positive	260 (81)	159 (76)	101 (89)				

a chi-square test.

b logistic regression.

**Figure 1:**
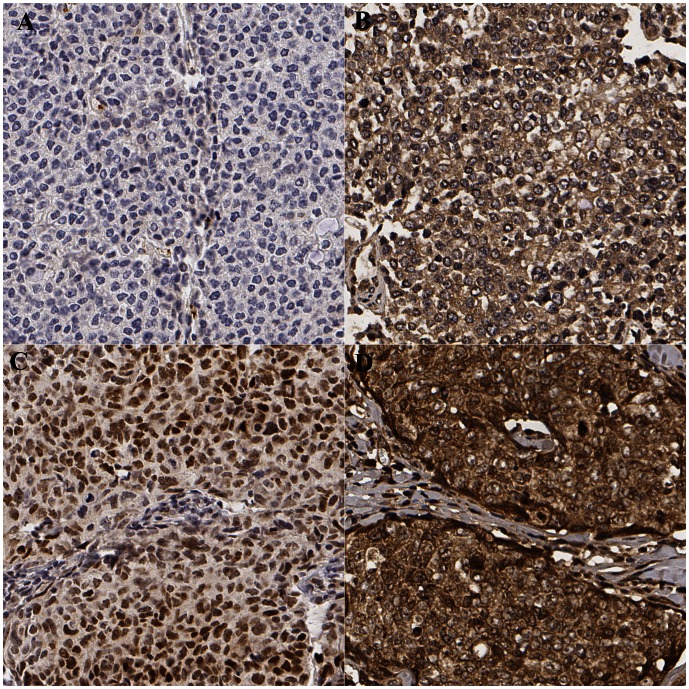
Examples of DDX3 and HIF-1α staining. Breast cancer photomicrographs are taken at 20X. A. low HIF-1α expression (0%); B. low DDX3 expression (1), same patient as in A; C. high HIF-1α expression (90%); D. high DDX3 expression (3), same patient as in C.

HIF-1α overexpression correlated with expression of CAIX, GLUT1, EGFR, HER2, Akt1, FER kinase, ERα, FOXO4, TfR, c-Met as expected (data not shown). Strong cytoplasmic DDX3 expression was associated with overexpression of the master regulator of the hypoxia response HIF-1α (OR = 2.83; p<0.001) (figure 1) and its downstream proteins GLUT1 (OR = 2.36; p = 0.001) and CAIX (2.39; p = 0.012). In logistic regression, HIF-1α (OR = 2.52; p = 0.001), GLUT1 (OR = 1.94; p = 0.021), CAIX (OR = 2.23; p = 0.042) predicted cytoplasmic DDX3 levels independently.(table 2).

The HIF-1α transcription regulators HER2 (OR = 3.04; p = 0.001), EGFR (OR = 2.01; p = 0.013) and Akt1 (83% vs. 95%; p = 0.001) were correlated with DDX3 expression. Association of EGFR and HER2 with DDX3 was dependent on HIF-1α.(table 3).

**Table 3:** Expression of DDX3 in relation to regulators of HIF-1α.

			Cytoplasmic DDX3						Correction for HIF-1α		
		*N* (%)	Low (%)	High (%)	OR	p value^a^			OR	p value^b^	
		366	239	127							
EGFR	negative	299 (83)	204 (86)	95 (76)	2.01	0.013			1.61	0.134	
	positive	62 (17)	32 (14)	30 (24)							
HER2	negative	325 (89)	222 (93)	103 (81)	3.04	0.001			1.88	0.092	
	positive	41 (11)	17 (7)	24 (19)							
p53	negative	80 (81)	45 (82)	35 (80)	1.16	0.802			1.42	0.571	
	positive	19 (19)	10 (18)	9 (20)							
COMMD1	low	23 (29)	17 (41)	6 (15)	3.90	0.013			5.45	0.006	
	high	57 (71)	24 (59)	33 (85)							
FER kinase	low	203 (57)	161 (70)	42 (34)	4.49	<0.001			4.10	<0.001	
	high	152 (43)	70 (30)	82 (66)							
PIN1	low	58 (71)	32 (78)	26 (63)	2.05	0.225			1.73	0.305	
	high	24 (29)	9 (22)	15 (37)							
			**Cytoplasmic DDX3**						**Cytoplasmic DDX3**		
		***N***	**Low**	**High**	**p value** c			***N***	**Low**	**High**	**p value** d
Akt1		80	83%	95%	0.001			75	86%	95%	0.026
FOXO4		75	30%	16%	0.035			61	21%	17%	0.600

a chi-square test.

b logistic regression.

c student’s t-test.

d ANCOVA.

Proteins known to regulate HIF-1α in a PI-3K/AKT independent fashion were associated with DDX3 as well; COMMD1 (OR = 3.90; p = 0.013), FER kinase (OR = 4.49; p<0.001), FOXO4 (30% vs. 16%; p = 0.035), but not p53 (OR = 1.16; p = 0.802) and PIN1 (OR = 2.05; p = 0.225).(table 3). Logistic regression indicated a HIF-1α independent relation between cytoplasmic DDX3 on the one hand and COMMD1 (OR = 5.45; p = 0.006) and FER kinase (OR = 4.10; p<0.001) on the other.(table 3).

DDX3 was further associated with ERα (OR = 0.48; p = 0.005), E-cadherin (OR = 2.84; p = 0.005), TfR (OR = 2.77; p<0.001), c-Met (OR = 1.72; p = 0.042) and FOXO3A (83% vs. 94%; p = 0.021). (table 4) After correction for HIF-1α expression, E-cadherin (OR = 2.91; p = 0.009), TfR (OR = 2.01; p = 0.007) and FOXO3A (78% vs. 95%; p = 0.007) were still associated with DDX3.(table 4). Table 5 shows the Pearson correlation analysis results.

**Table 4:** Expression of DDX3 in relation to various other hypoxia induced proteins.

		*N* (%)	Low (%)	High (%)	OR	p value^a^		OR	p value^b^	
		366	239	127						
ERα	negative	81 (22)	42 (18)	39 (31)	0.48	0.005		0.67	0.166	
	positive	285 (78)	197 (82)	88 (69)						
E-cadherin*	low	48 (17)	38 (23)	10 (9)	2.84	0.005		2.91	0.009	
	high	227 (83)	130 (77)	97 (91)						
p21	low	46 (46)	28 (51)	18 (41)	1.50	0.418		1.26	0.625	
	high	53 (54)	27 (49)	26 (59)						
TfR	low	221 (63)	161 (72)	60 (48)	2.77	<0.001		2.01	0.007	
	high	128 (37)	63 (28)	65 (52)						
c-Met	negative	264 (77)	181 (81)	83 (71)	1.72	0.042		1.62	0.096	
	positive	77 (23)	43 (19)	34 (29)						
			**Cytoplasmic DDX3**					**Cytoplasmic DDX3**		
		***N***	**Low**	**High**	**p value** c		***N***	**Low**	**High**	**p value** d
p27		99	42%	44%	0.683		77	40%	45%	0.507
FOXO3A		86	83%	94%	0.021		71	78%	95%	0.007
Notch1		305	63%	55%	0.064		282	61%	56%	0.299

a chi-square test.

b logistic regression.

c student’s t-test.

d ANCOVA.

* in ductal breast cancer.

TfR = Transferrin receptor.

**Table 5:** DDX3 correlations with the most important hypoxia related proteins.

	*N*	r^a^	p value
HIF-1α	322	0.276	<0.001
GLUT1	313	0.186	0.001
CAIX	322	0.136	0.015
HER2	366	0.185	<0.001
ERα	366	-0.132	0.011

a Pearson correlation coefficient.

## Discussion

The aim of this study was to investigate the relation between DDX3 and the hypoxic response in human breast cancer in the light of *in vitro* results pointing to regulation of DDX3 by HIF-1α. We indeed show a positive correlation between HIF-1α and DDX3 overexpression in a large series of human breast cancer cases, as well as an association between DDX3 overexpression and various other hypoxia related proteins.

However, we have established a correlation between DDX3 overexpression and nuclear HIF-1α overexpression which supports the direct regulation of DDX3 by HIF-1α found *in vitro* [21], but this is obviously no more than an association at this point no proof for a causal relationship. Immunohistochemistry has some limitations like being inherently a more qualitative than quantitative method, and semiquantitative scoring and dichotomization with non-optimal reproducibility. To compensate for these issues we standardized the IHC procedure, used control tissue throughout, scored three samples per patient, studied a large cohort of breast cancer patients and results obtained from dichotomized parameters were confirmed by correlation analysis for the most important parameters with the DAKO score of DDX3 (table 5). Patient features in this study corresponded with known clinicopathological characteristics in breast cancer (table 1) [65].

Furthermore, DDX3 correlated with EGFR, HER2, FOXO4, ERα and c-Met in a HIF-1α dependent way. Also, we found a positive correlation with COMMD1, FER kinase, Akt1, E-cadherin, TfR and FOXO3A independent of HIF-1α. COMMD1 down regulates HIF-1α by competition with HSP90β [41], or down regulates the transcriptional activity of HIF-1α [40]. However, we could not detect an association with COMMD1 and HIF-1α expression or its downstream targets: E-cadherin, TfR, p21, p27 or c-Met. Nonetheless, COMMD1 correlates with DDX3 independent of HIF-1α.

FER kinases help cells to withstand stress, including hypoxia, via up regulation of HIF-1α [42]. We found a strong relation with FER kinase with both HIF-1α and DDX3. After correction for the effect FER kinase has on HIF-1α, a strong relation between FER kinase and DDX3 remained, implying a HIF-1α dependent and independent relation.

DDX3 was shown to down regulate E-cadherin [12], but in the present study we show a positive correlation, for which we have no obvious explanation. TfR is also under transcriptional control of HIF-1α [47]. TfR is overexpressed in many cancers, which could be attributed to the increased need for iron as a cofactor of the ribonucleotide reductase enzyme involved in DNA synthesis of rapidly dividing cells. Thus, the HIF-1α independent relation between DDX3 and TfR corroborates previous reports on the oncogenic properties of DDX3. Nuclear expression of FOXO3A in breast cancer is associated with anti-apoptotic signaling via Akt1, an aggressive phenotype and poor survival [66]. In response to hypoxia, FOXO3A accumulates in a HIF-1α dependent way to inhibit HIF-1α induced apoptosis [48]. Although we did not find a relation between HIF-1α and FOXO3A we did find a relation between FOXO3A and DDX3, independent of HIF-1α and Akt1. Perhaps DDX3 and FOXO3A function in a concerted survival response after stress stimuli.

EGFR, HER2 and Akt1 regulate HIF-1α transcription in a PI-3K/AKT dependent fashion[30]–[33]. As expected, the positive correlation between DDX3, on the one hand and EGFR and HER2 on the other was HIF-1α dependent. Moreover, HER2 and EGFR regulation of HIF-1α was Akt1 dependent. Furthermore, in 93% of patient samples with high expression of DDX3 and HIF-1α, Akt1 was highly expressed of which 71% of these patients also had EGFR or HER2 overexpression. This fits with a concerted HER2/EGFR-Akt1-HIF-1α-DDX3 pathway, which is consistent with previous reports [21], [34].

In conclusion, ten of eighteen proteins analyzed by IHC showed a similar HIF-1α related effect as described in the literature. All these ten HIF-1α related proteins were associated with expression of DDX3 as well, indicating an important role for DDX3 in the hypoxia response via HIF-1α, and underlying the oncogenic role of DDX3. Since hypoxic tumor regions are typically resistant to current therapy [27], this emphasizes the potential of DDX3 inhibitors, perhaps in combination with HER2 and/or EGFR inhibitors.
